# Contextualized Filtering for Shared Cyber Threat Information

**DOI:** 10.3390/s21144890

**Published:** 2021-07-18

**Authors:** Athanasios Dimitriadis, Christos Prassas, Jose Luis Flores, Boonserm Kulvatunyou, Nenad Ivezic, Dimitris A. Gritzalis, Ioannis K. Mavridis

**Affiliations:** 1 Department of Applied Informatics, University of Macedonia, 156 Egnatia Str., 54636 Thessaloniki, Greece; asdimitriadis@uom.edu.gr (A.D.); prassas@uom.edu.gr (C.P.); 2 Associate, Engineering Laboratory, National Institute of Standards and Technology, 100 Bureau Drive, Gaithersburg, MD 20899, USA; 3 Industrial Cybersecurity, IKERLAN Technology Research Center, Basque Research and Technology Alliance (BRTA), P.J.M. Arizmendiarrieta 2, 20500 Arrasate/Mondragón, Spain; jlflores@ikerlan.es; 4 Engineering Laboratory, National Institute of Standards and Technology, 100 Bureau Drive, Gaithersburg, MD 20899, USA; boonserm.kulvatunyou@nist.gov (B.K.); nenad.ivezic@nist.gov (N.I.); 5 Department of Informatics, Athens University of Economics and Business (AUEB), 10434 Athens, Greece; dgrit@aueb.gr

**Keywords:** cyber threat information sharing, actionable threat information, filtering, business process context

## Abstract

Cyber threat information sharing is an imperative process towards achieving collaborative security, but it poses several challenges. One crucial challenge is the plethora of shared threat information. Therefore, there is a need to advance filtering of such information. While the state-of-the-art in filtering relies primarily on keyword- and domain-based searching, these approaches require sizable human involvement and rarely available domain expertise. Recent research revealed the need for harvesting of business information to fill the gap in filtering, albeit it resulted in providing coarse-grained filtering based on the utilization of such information. This paper presents a novel contextualized filtering approach that exploits standardized and multi-level contextual information of business processes. The contextual information describes the conditions under which a given threat information is actionable from an organization perspective. Therefore, it can automate filtering by measuring the equivalence between the context of the shared threat information and the context of the consuming organization. The paper directly contributes to filtering challenge and indirectly to automated customized threat information sharing. Moreover, the paper proposes the architecture of a cyber threat information sharing ecosystem that operates according to the proposed filtering approach and defines the characteristics that are advantageous to filtering approaches. Implementation of the proposed approach can support compliance with the Special Publication 800-150 of the National Institute of Standards and Technology.

## 1. Introduction

Accurate and timely analysis of cyber-attacks is crucial for effective prevention, detection, and response [1]. This becomes quite challenging, especially in the context of complex information and communication technology infrastructures that have resulted in an increased number of vulnerabilities. The industrial internet of things paradigm has exacerbated the situation, making traditional security approaches become inappropriate or considerably challenged [2]. On the other side, threat actors are becoming more intelligent and incredibly strategic, utilizing advanced and continuously evolving attack techniques. The targets of cyber-attacks can range from small–medium enterprises (SME) to critical infrastructure services, putting a large number of sectors at risk. Some examples are the cases of WannaCry [3] and Petya [4] ransomwares, as well as the case of Mirai Botnet [5], all of which spread over or affected many private and public sectors.

To cope with this threat landscape, organizations are constantly trying to mature their security capabilities. Nevertheless, it is next to impossible to achieve security objectives with an organization’s sole effort [6]. Organizations need to collaborate by sharing cyber threat information (CTI) [7]. According to a SANS survey in [8], almost 81% of organizations that were sharing CTI answered that CTI had improved their security and response to threats. CTI can be shared in the form of a CTI Product (CTIP), which is a message containing threat-related information that can be used for controlling ongoing, imminent, or future threats. To support organizations with CTIP sharing, a lot of CTIP repositories have been developed, such as the AlienVault OTX [9]. A CTIP repository allows organizations to publish and consume CTIPs; that is, it enables CTIP sharing. The need for CTIP sharing towards developing collaborative security is also embraced by leading cybersecurity organizations such as Lockheed Martin, the Information Technology Laboratory (ITL) of the National Institute of Standards and Technology (NIST), and the European Parliament. In particular, Lockheed Martin proposed an information sharing approach that utilizes the exchange of knowledge derived from past attacks in the form of Indicators of Compromise (IoC) [10]. The ITL of NIST published guides regarding the cooperation and sharing of security-related information between organizations in the United States [11]. The European Parliament and the Council of 6 July 2016 issued the Networks and Information Systems Directive to support and facilitate the strategic cooperation and exchange of information between organizations [12].

CTIP sharing comes with a lot of security-related benefits, but also with numerous challenges [11]. According to the NIST’s guides on CTI sharing, one challenge of great importance is the ability to filter shared CTIPs [11]. Filtering allows organizations to retrieve and process only the CTIPs that are relevant to them. A CTIP is relevant to an organization when its contained threat-related information relates to the organization’s context (e.g., location, domain, business process). A relevant CTIP is expected to be actionable to the organization consuming it [11,13,14]. A CTIP is actionable when the utilization of its content (i.e., the threat-related information that it contains) leads to a decision or an action to control an ongoing, imminent, or a future threat. The necessity of filtering to avoid overload and “distilling the signal from the noise” has been extensively pointed out in the literature [13,14,15,16,17,18]. The lack of filtering overwhelms organizations’ processing capabilities [11] and demands expertise for narrowing down shared CTIP manually [14]. The overload of CTIP causes the organizations’ reluctance to participate in threat information sharing ecosystems, particularly in the case of SMEs, that represent 99% of all organizations in Europe [19] and in the United States [20].

Commonly used filtering approaches include keyword searching and domain tagging. Keyword searching concerns the filtering of CTIPs based on specific key terms (e.g., phishing, Windows, WannaCry, USA). Keyword searching is offered by most CTIP repositories [14]. Domain tagging concerns the categorization of shared CTIPs into domains, such as finance and education, with the utmost goal of presenting them to the organizations belonging to the same domain [21]. Keyword searching is labor-intensive, error-prone, and demands expertise in order to define the proper keywords [13,14,15], while the use of domain tagging is insufficient since current approaches only classify CTIP into a few arbitrary, high-level domains that are not sufficiently specific. Recent research efforts in CTIP filtering have suggested the exploitation of business information, which is a domain knowledge that either already exists or can be created by personnel with domain knowledge (and then used by the security personnel) [13,22]. In particular, the contextual business information has been singled out as promising [11,13,23]. Using contextual business information provides improvements over domain tagging by eliminating the arbitrary domains, enabling finer grain categorization. Few results, however, have been generated insofar. As such, it is without doubt that there is a need to develop a new filtering approach based on contextual business information.

In this paper, we propose a novel, automated, contextualized filtering approach for shared cyber threat information. To this end, the contextual information of business processes is utilized to define the CTIP’s context (i.e., the context to which the threat-related information is related) as well as the organization’s context. Filtering is performed based on the equivalence between the forenamed contexts. In doing so, organizations receive only the CTIPs whose context is equivalent to theirs. In other words, they will receive CTIPs that are likely actionable to them. The proposed approach should be implemented at the server-side (i.e., within the CTIP repository) to foster trustworthiness, privacy, and ease organizations from the filtering process, allowing them to focus their efforts on the consumption of the received CTIPs only. To promote the adoption of the proposed approach, the paper also outlines industry standards and open-source data exchange protocols that can be used to establish a CTIP sharing ecosystem. The use of standards promotes the use of shared semantics (e.g., as opposed to arbitrary domains), making the filtering more reliable. The proposed approach deals with the challenge on CTI sharing identified in [11], in that organizations should find an effective way to identify the CTIPs that are applicable (i.e., actionable) to their environments

The rest of the paper is organized as follows: Section 2 presents the background necessary for presenting the proposed approach. Section 3 details the proposed filtering approach. Section 4 presents the proposed equivalence measurement between two contexts. In Section 5, the applicability of the proposed approach is demonstrated through a proposed CTIP sharing ecosystem. Section 6 discusses the related work and reports the results, and Section 7 concludes the paper.

## 2. Background

This section describes concepts that are essential in order to understand the subsequent discussions and the proposed approach.

### 2.1. Structured Threat Information Expression Language

Cyber threat information should be represented in a standardized, structured format in order to enable CTI sharing automation [11]. This need resulted in the development of expression languages. STIX, also known as Structured Threat Information eXpression (STIX) language [24], is commonly used [25,26]. In Europe, STIX was recognized as an information standard by the Commission Implementing Decision 2017/2288 of 11 December 2017 [27]. STIX was initially developed by the United States government and MITRE. Currently, it is maintained by the Organization for the Advancement of Structured Information Standards (OASIS) [28].

STIX is a structured language used for creating, sharing, and consistently consuming CTIPs [29]. In the current STIX 2.1 version, CTIPs are formatted using JavaScript Object Notation (JSON). A CTIP ties together a diverse set of STIX objects along with their descriptive relationships. STIX objects are instances of STIX Domain Objects (SDO) or STIX Cyber-observable Objects (SCO), while the relationships are instances of STIX Relationship Objects (SRO) [29]. An example of a single STIX object CTIP is the report of a single malware, while an incident reporting would require a multi-object CTIP. There exist various SDOs for representing different concepts in the CTI domain. For instance, the Malware SDO for representing malicious software, Indicator SDO for representing imminent or in-progress attack, and Identity SDO for representing an individual or organization. There are also numerous SCOs that can be used within SDOs for providing more technical details related to host- or network-based artifacts, such as IP addresses and URLs. As for the SROs, there are two types of them: the Relationship SRO and the Sighting SRO. The former represents the relationship among SDOs and SCOs. The Sighting SRO allows for an organization to communicate that a STIX object contained in a shared CTIP was observed in its environment. In other words, Sighting SRO indicates that the CTIP was actionable in the observed environment.

**Figure 1:**
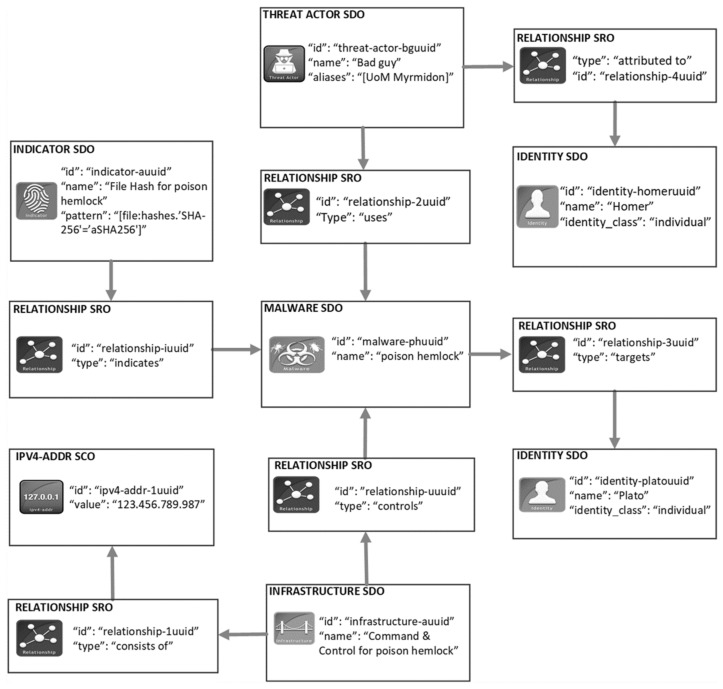
A graphical representation of a cyber threat information product in the STIX language.

Figure 1 depicts a graphical example of a CTIP in the STIX language (for simplicity’s sake, only parts of the SDO and SRO details are depicted). This example is an incident report describing that “Homer” targeted “Plato” using the malware “poison hemlock” which connects to the server operating in the IP address “123.456.789.987”. The Indicator SDO reveals that a system is compromised by the malware “poison hemlock”. Finally, the creator of the CTIP is “Plato”—the first victim of this malware.

**Figure 2:**
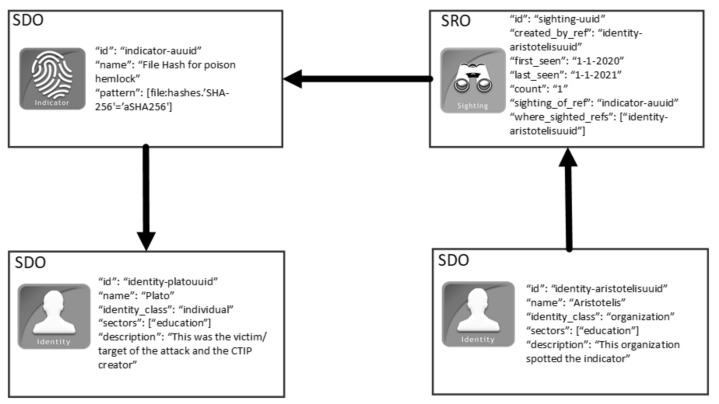
An illustrative example of a Sighting STIX Relationship Object.

As already mentioned, a Sighting SRO allows organizations to communicate back to the CTIP sharing community that an SDO or SCO of a CTIP was also seen in their environments. According to OASIS, it is vital for the community to be aware that an SDO or SCO of a CTIP was also spotted elsewhere [30]. Figure 2 depicts an example of a Sighting SRO that relates to the Indicator SDO of Figure 1. This Sighting SRO communicates that the Indicator SDO of the above example was spotted in the environment of “Aristotelis”.

### 2.2. Business Process Context

The proposed filtering approach utilizes the context of business processes, called Business Process Context (BPC), towards achieving filtering. The BPC representation has been proposed by Ivezic et al. [31] and it is based on industry standards, such as ISO 3166 that provides the codes for the representation of names of countries as well as their subdivisions. In [31], the BPC is used to set the context of message specifications that are exchanged in systems integration cases. Those boilerplate message specifications are huge since they contain all data elements to cover many integration cases in many contexts. Therefore, much effort is required to determine the data elements of a message specification that are applicable to a specific integration case, which is the context indicating where a boilerplate message specification is used. With BPC, message specifications can be profiled according to the intended integration case. In other words, the BPC specifies which data elements of the message specification are needed in a specific integration case.

**Figure 3:**
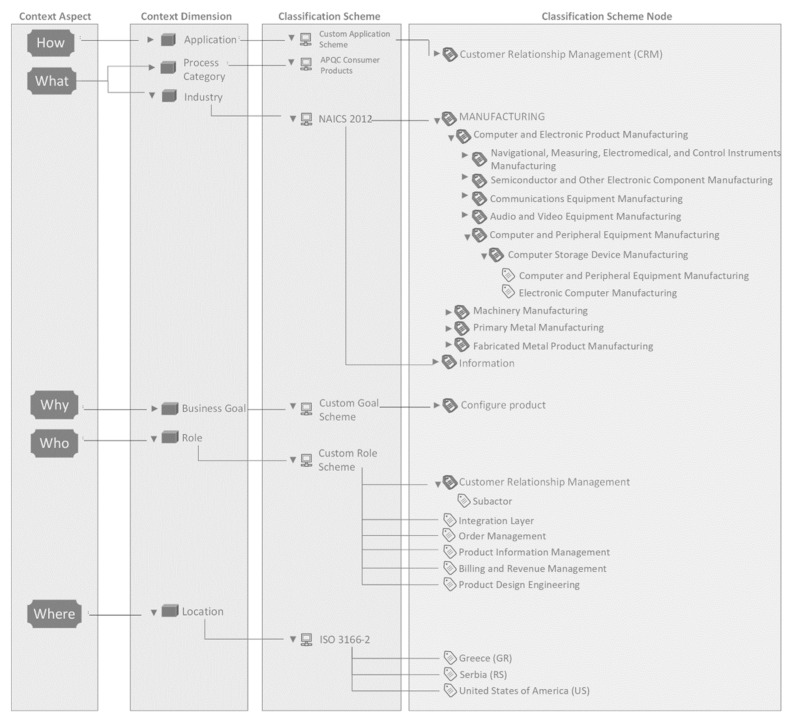
An illustrative example of the Business Process Context structure.

Six Context Aspects (CAs) were used in [31] to describe an integration case, including Why, When, Who, Where, What, and How. Each CA is specified by multiple Context Dimensions. For example, the Context Dimension “Location” specifies the Context Aspect “Where”. The allowed set of values for each Context Dimension is derived from a Classification Scheme. For instance, the Classification Scheme “ISO 3166” sets the range of values for the Context Dimension “Location” (i.e., CA “Where”). These values are called Classification Scheme Nodes and they are organized in a specialization hierarchy. That is, low-level nodes (child nodes) are grouped under higher-level nodes (parent nodes). Figure 3 depicts how all the forenamed notions are linked together (due to space limitation, only part of Context Dimensions, Classification Schemes, and Classification Scheme Nodes are depicted). It should be noted that the CA “When” is not used in [31].

**Figure 4:**
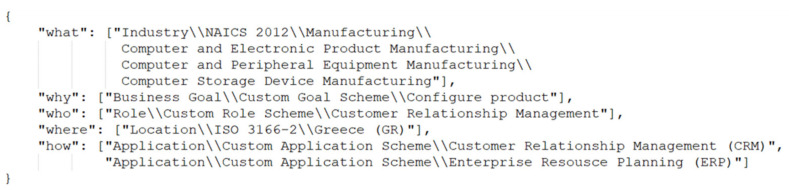
An example of one BPC of a business process serialized in JSON.

In the proposed approach, the BPC is serialized in JSON. An example of one BPC of a business process serialized in JSON is depicted in Figure 4. In this paper, a path syntax is proposed to represent each CA value. The path separator is the double-backslash. The first element in the path shall be the Context Dimension for the corresponding CA, the second element is the Classification Scheme, and the rest are Classification Scheme Nodes following the hierarchical path. All elements in the context path are represented as string values. A CA can contain more than one value. For instance, the context aspect “How” in Figure 4 contains two values related to the applications involved in the corresponding business process. If a CA value points to the deepest leaf node in the hierarchy, the CA is considered fully defined. With respect to the context schemes used and for illustration in this paper, the CAs “What”, “Why”, “Who”, “Where”, and “How” are fully defined if their context path contains 7, 3, 4, 3, and 3 elements, respectively. For instance, based on the context scheme “ISO 3166-2”, the context path of the CA “Where” can be “Location\\ISO 3166-2\\Greece (GR)”. This context path contains 3 elements. In the example in Figure 4, the CAs “Why”, “Where”, and “How” are fully defined, while the CAs “What” and “Who” are partially defined.

## 3. Proposed Contextualized Filtering Approach

Contextualized filtering is proposed to be performed based on the equivalence of the organization’s and CTIP’s context. In case these two contexts are equivalent, the CTIP is considered relevant and can be pushed to the corresponding organization. In this way, organizations receive CTIPs that are expected to be actionable to them.

To define the organization’s and CTIP’s context, the BPC is utilized. The organization’s context consists of the BPCs of all its business processes. The CTIP’s context is composed of the BPCs of all business processes that are related to the CTIP. Each CTIP is related to the business processes that were affected or targeted by the threat information described in this CTIP. For instance, a CTIP that contains a Malware SDO is related to the business process wherein the malware was found.

To realize the proposed approach, the BPC is integrated into the CTI domain via a user-defined STIX object (i.e., custom STIX object). For that, a BPC SDO is defined to describe one or more BPCs. The BPC SDO consists of a set of CAs, as well as STIX metadata (i.e., ID, version). Each CA contains the corresponding CA values of all described BPCs. All STIX objects can have a context except SROs. SROs cannot have a context assigned since they do not convey any CTI concept. They are only used to link SDOs and SCOs together. The context of a STIX object is defined by linking a BPC SDO to that STIX object.

**Figure 5:**
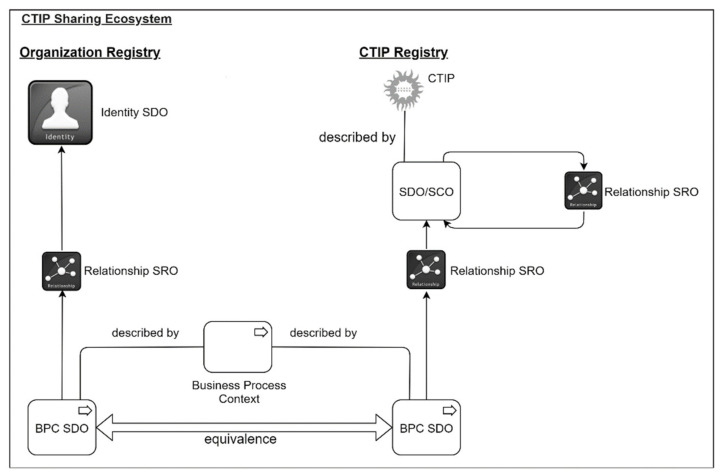
Business Process Context representation in a CTIP sharing ecosystem.

Identity SDO is used to describe an organization in a CTIP according to STIX. In the proposed approach, it is also used to describe organizations registered to a CTIP repository. In this vein, the organization’s context is defined by a BPC SDO linked to the Identity SDO in the Organization Registry shown in Figure 5. The CTIP’s context is the combination of the BPC SDOs belonging to all CTIP’s SDOs and SCOs. CTIPs are in the CTIP registry. Both Organization and CTIP Registry are in the CTIP repository.

Filtering is performed based on the equivalence between organization’s and CTIP’s context, i.e., between two BPC SDOs. One BPC SDO is linked to an Identity SDO in the Organization Registry and the other is linked to a STIX object describing a CTIP in the CTIP Registry. The equivalence between two BPC SDOs (i.e., BPCs) is based on the measure described in the next section. This measure is based on the CA values of BPC SDOs, and STIX metadata is not considered (e.g., the STIX version). When there is an equivalence with any STIX object of a CTIP, the whole CTIP is pushed to the organization (e.g., the report in Figure 1). This least equivalence approach aims at minimizing false negatives, which means that an organization does not receive a CTIP even if there is equivalence with at least one of the CTIP’s objects. Intuitively, it may seem that organizations might receive higher rates of false positives. Nevertheless, this is rarely the case since organizations will receive CTIPs that contain at least one STIX object with equivalent context. Therefore, at least this STIX object is actionable to the receiving organization. In the best scenario, the whole CTIP will be actionable and will reveal threats to other business processes that organizations might have omitted to describe in their BPC SDO, either intentionally or accidentally.

Filtering should cover cases where the equivalence between two BPC SDOs is not absolute, i.e., their CA values are all not identical. The reasons are that organizations might not have provided their complete context, or they want to perform risk estimation or contextual changes (i.e., BPC changes). Furthermore, filtering should take into account preferences where organizations might be interested in some CAs more than others (e.g., the CA “Where”). For these reasons, the proposed approach introduces a threshold and weights to CAs. Weights allow organizations to set CA preferences, while the threshold allows organizations to receive CTIPs only if their equivalence measures are above that threshold level. Both threshold and weights can be adjusted by organizations to their needs.

**Figure 6:**
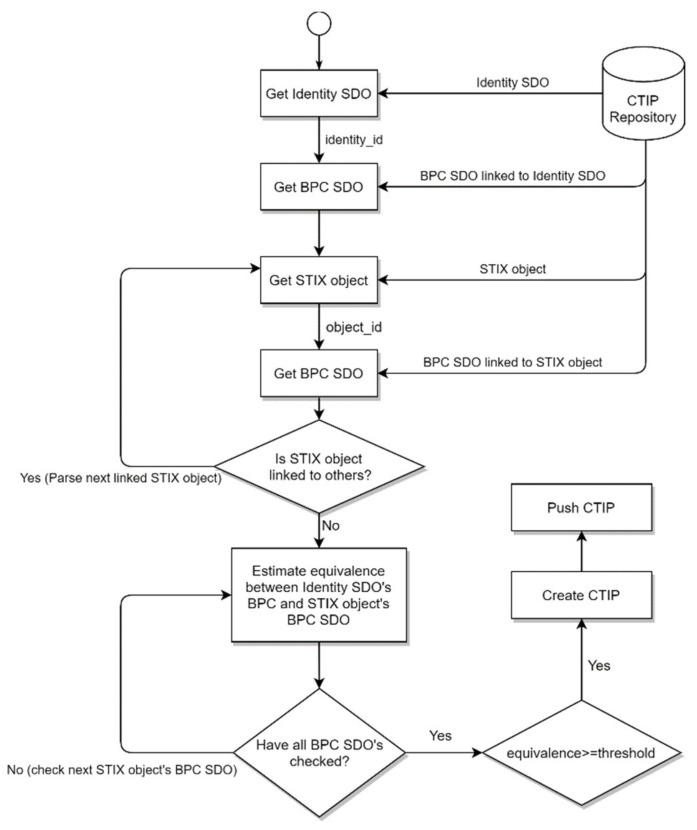
Flowchart diagram representing the workflow of the proposed filtering approach.

The flowchart diagram in Figure 6 shows the filtering approach, as described above. As a start, a default value is given to the threshold and balanced weights are given to CAs (i.e., all CAs play the same important role in the equivalence measurement). The proposed filtering approach is triggered whenever a new CTIP is published or an organization becomes a member of a CTIP sharing ecosystem. In the case of publishing a new CTIP, filtering is performed for each organization. To do so, the BPC SDO in the Organization Registry is parsed and checked against the BPC SDO of all STIX objects of this newly published CTIP. In case an organization becomes a new member in a CTIP sharing ecosystem, all STIX objects in the CTIP Registry are checked, as the organization needs to be situationally aware of all existing threats against its environment.

## 4. Equivalence Measurement

**Figure 7:**
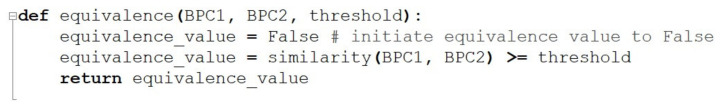
Method for estimating the equivalence between two Business Process Contexts.

As depicted in the code in Figure 7, two BPCs (BPC1 and BPC2) are equivalent if their similarity (bpc_similarity) is above a specific threshold. Equivalence is a Boolean value, with True representing that two BPCs are equivalent and False the opposite. The bpc_similarity and threshold values, which are explained below, range from 0 to less than 1 (inclusive of 0, but not 1).

The bpc_similarity, shown in Equation (1), estimates the degree to which two BPCs (BPC1 and BPC2) are alike. The bpc_similarity is an increasing function, i.e., the greater its value, the greater the degree to which the BPCs are alike. The bpc_similarity value ranges from 0 to less than 1 (i.e., it is asymptotic to 1) and it is based on the similarities of the CA of two BPCs (i.e., $ca\_similarity\;\left(BPC1CA_{i},BPC2CA_{i}\right)$).

(1) $$bpc\_similarity\left(BPC1,BPC2\right)=\overset{n}{\underset{i=1}{\sum }}weight_{i}*ca\_similarity\left(BPC1CA_{i},BPC2CA_{i}\right)$$

The variable n represents the maximum number of the CAs that can be used to define the BPC. In case of defining BPC utilizing [31], *n* is 5 since the CA “When” is not used. The weights (i.e., $weight_{i}$) define the role of each CA in calculating the bpc_similarity and can be customized by each organization in the Organization Registry. The sum of all weights must be equal to 1 and every weight must be within the range [0,1]. A CA can contain more than one value. In this case, the bpc_similarity function uses the appropriate CA value that maximizes the bpc_similarity value, and all other values of this CA are ignored. For example, the BPC1 and BPC2 described below, respectively contain one and two values per their CA “What”. In this example, the bpc_similarity function will use the first value of the BPC2′s CA, “Industry\\NAICS 2012\\Manufacturing”, since it is identical to the one of BPC1′s CA. The reason for selecting this CA value is that it maximizes the bpc_similarity value; indeed, it provides a greater similarity than using the second value.

- BPC1′s CA “What”: “Industry\\NAICS 2012\\Manufacturing”
- BPC2′s CA “What”: [“Industry\\NAICS 2012\\Manufacturing”, “Industry\\NAICS 2012\\Information”]

The ca_similarity, shown in Equation (2), estimates the degree to which two CA values ($CA_{1},\;CA_{2}$) are alike. The CA values are represented by context paths, as mentioned in Section 2.2. The ca_similarity is calculated based on the matches at the element level. Each element in the first context path is compared to its corresponding element of the second context path in their respective order. If both elements represent the same string value (i.e., string comparison), the process continues, comparing the next element and so forth. The process continues until all elements in the shorter path are compared or until a difference is found. In the first case, p is assigned with the position of the last element + 1. In the latter case, p is assigned with the position of the two elements that are not the same. p is then used for calculating the ca_similarity, as shown in Equation (2):

(2) $$ca\_similarity\left(CA_{1},\;CA_{2}\right)=1-\frac{1}{p}$$

**Figure 8:**
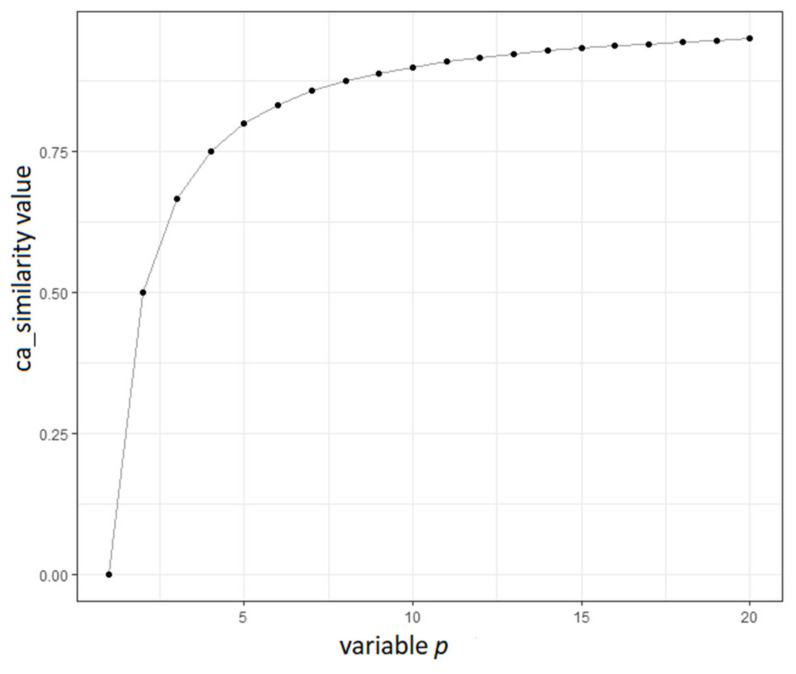
Plot of the ca_similarity value against the variable p.

Figure 8 plots the ca_similarity against the variable p. The latter variable is the key parameter to differentiate cases with various context path lengths. The greater the path, the greater the ca_similarity and, consequently, the bpc_similarity. It should be noted that the ca_similarity value is asymptotic to 1, which causes the bpc_similarity value to be asymptotic to 1 as well. This was developed on purpose to allow for future additions in the CAs and more depths in the hierarchical levels that are described in Section 2.2. It should be noted that the application of machine learning techniques could result in a linear plot without providing better results against the proposed ca_similarity equation. As depicted in the plot of Figure 8, ca_similarity indeed differentiates cases efficiently, regardless of the variable *p*.

The threshold, which is used in Equation (1), sets the lower-bound bpc_similarity value, beyond which two BPCs are considered equivalent. The threshold ranges from 0 to the maximum bpc_similarity value. When the threshold is set to 0, all BPCs are considered equivalent. When it is set to the maximum bpc_similarity value, equivalence is granted only when two BPCs are identical. Threshold can be changed by organizations according to their preferences. The bpc_similarity value is at its maximum for a given weight allocation when all CA values are fully defined and matched. For example, when utilizing the BPC defined in [31], maximum bpc_similarity between two BPCs occurs when their CAs “Where”, “What”, “Who”, “Why”, and “How” consist of 3, 7, 4, 3, and 3 elements respectively, and their values are exactly the same. Based on Equation (2), their ca_similarity values are 0.75, 0.875, 0.8, 0.75, and 0.75, respectively. According to Equation (1), the maximum bpc_similarity value if given equal weights is 0.785, as shown in Equation (3):

(3) $$bpc\_similarity\left(bpc1,\;bpc2\right)=\overset{5}{\underset{i=1}{\sum }}weight_{i}*ca\_similarity(BPC1CA_{i},BPC2CA_{i})\;=0.2*\left(0.75+0.875+0.8+0.75+0.75\right)=0.785$$

## 5. Proposed CTIP Sharing Ecosystem Implementing the Proposed Filtering Approach

**Figure 9:**
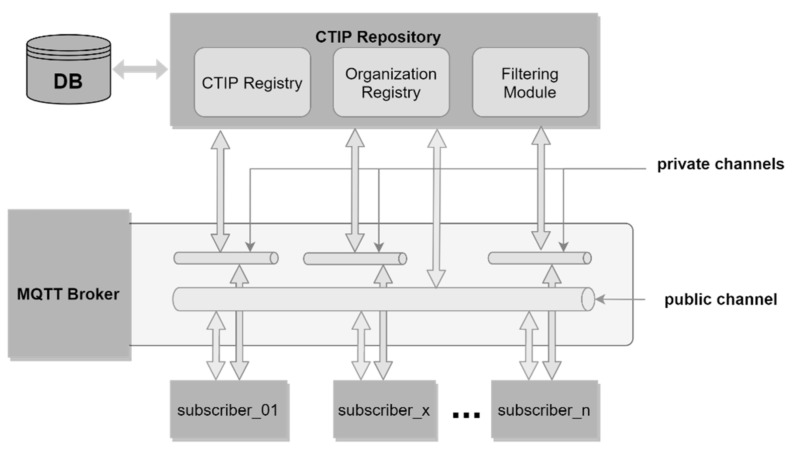
Architecture of the proposed CTIP sharing ecosystem.

In this section, a CTIP sharing ecosystem that can implement the filtering approach effectively is proposed, and its architecture is depicted in Figure 9. The ecosystem includes the following components: a CTIP repository, a database, subscribers (i.e., the participating organizations), and a standard-based Message Queuing Telemetry Transport (MQTT) broker [32]. Opensource tools are available for the database and the MQTT broker and encourage widespread participation. In our lab implementation, mongoDB was used for the database, and Eclipse Mosquito was used for the MQTT broker [33].

The CTIP repository consists of the CTIP Registry, Organization Registry, and Filtering Module. CTIP Registry is responsible for storing and retrieving CTIPs to/from the database. The Organization Registry manages the subscribers’ information, including their communication channels, CA weights, threshold, Identity SDOs, and BPC SDOs. The CTIP Filtering Module is responsible for implementing the proposed filtering approach in order to push the CTIPs to the proper subscribers according to the equivalence measurement.

The communication between subscribers and the CTIP repository should take place via channels. To do so, an MQTT broker that implements a publish–subscribe messaging pattern via channels should be employed. An MQTT broker allows communication among subscribers via channels (in terms of the MQTT domain, a channel is called a topic). Once one is subscribed to a channel, they can receive everything that is published to this channel by someone else.

There should be two types of communication channels—public and private. The former involves one public channel and the latter several private channels. The public channel is used by the CTIP repository and all subscribers, while each private channel is used by the CTIP repository and a specific subscriber only. The public channel is used for subscription purposes. No other activity should be allowed there (i.e., publication of CTIPs). This enables the implementation of filtering. Otherwise, all CTIPs published to the public channel would be pushed to all subscribers automatically without having been filtered first. Private channels are used for exchanging CTIPs and subscribers’ information, such as its Identity SDO and BPC SDO. Exchanging subscribers’ information in private channels ensures that no one else except the CTIP repository will receive it. On the other hand, the exchange of CTIPs in private channels ensures the anonymity of subscribers’ actions related to the creation, publication, and consumption of CTIPs. Such a communication policy protects an organization’s reputation and encourages more participation.

The mechanism of the proposed CTIP sharing ecosystem is presented in five stages. The five stages are presented in the following five figures using UML behavioral diagrams. For simplicity’s sake, only the necessary information of the parameters of the exchanged messages is included.

**Figure 10:**
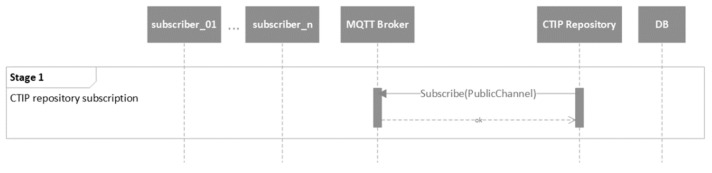
UML behavioral diagram of the CTIP repository subscription stage (stage 1).

The first stage is the CTIP repository subscription, as depicted in Figure 10. In particular, the CTIP repository subscribes through the “PublicChannel”. As already mentioned, the public channel should be used for subscription purposes only. For this purpose, the code of the MQTT broker can be modified to enforce such policy, but this is beyond the scope of this effort.

**Figure 11:**
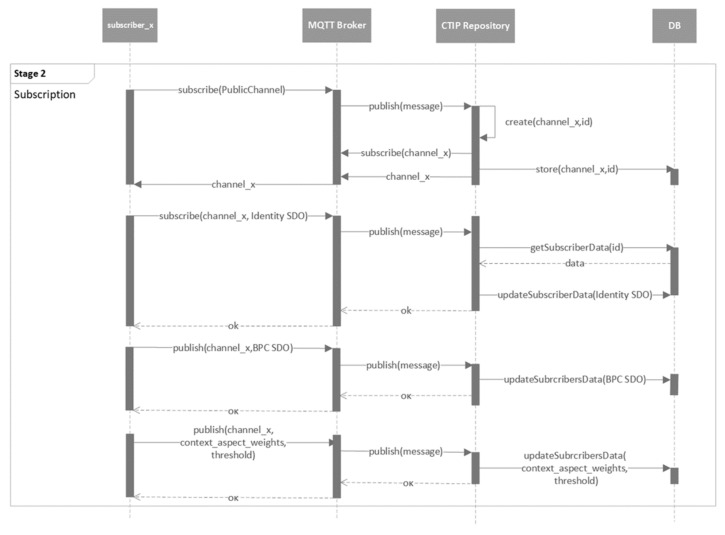
UML behavioral diagram of the organizations’ subscription stage (stage 2).

The next stage concerns the subscription of the organizations, as depicted in Figure 11. To do so, an organization (i.e., “subscriber_x) subscribes to “PublicChannel” first. Afterward, a private channel (e.g., “channel_x”) is created by the CTIP repository and the name of the channel is given back to the organization. Both the CTIP repository and this specific organization are subscribed to this “channel_x” (no one else should be allowed to subscribe to this channel). The name of the channel, however, is visible to all other organizations since it is sent via the public channel (i.e., “PublicChannel”). Digital certificates can be used to protect this information; however, it is beyond the scope of this paper. In each private channel, the corresponding subscriber can publish CTIPs as well as its Identity SDO, BPC SDO, the CAs weights, and the threshold.

**Figure 12:**
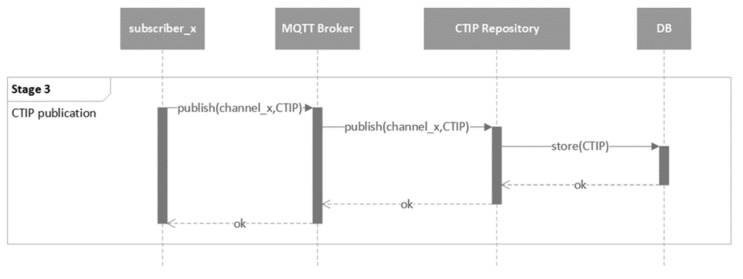
UML behavioral diagram of the CTIP publication stage (stage 3).

The next stage is the CTIP publication, as depicted in Figure 12. Subscribers publish CTIPs to the MQTT broker through their private channels, which are then forwarded to the CTIP repository through the same channel. Afterward, the CTIP repository stores CTIPs to the database.

**Figure 13:**
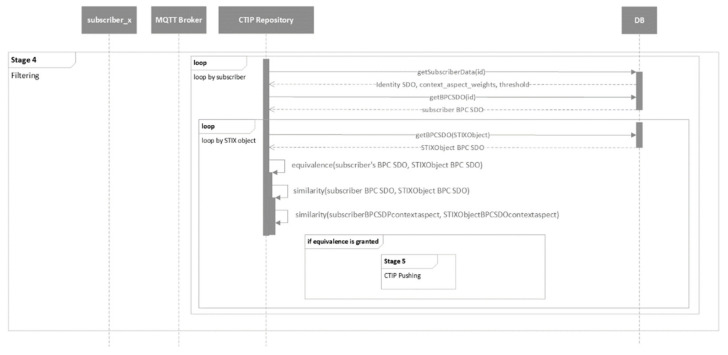
UML behavioral diagram of the filtering stage (stage 4).

Figure 13 presents the CTIP filtering stage. In this stage, the CTIP repository performs filtering, as detailed in Section 3 and depicted in Figure 5 and Figure 6. If equivalence is granted, the process moves to Stage 5, otherwise it continues for the next organization in the Organization Registry or STIX object in the CTIP Registry until all of them are checked.

**Figure 14:**
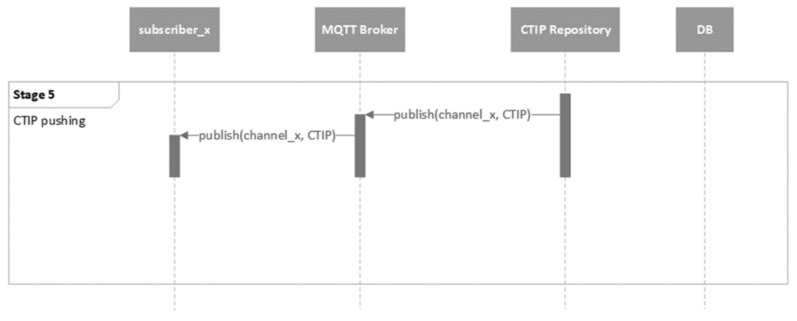
UML behavioral diagram of the CTIP pushing stage (stage 5).

The next stage is the CTIP pushing, as depicted in Figure 14. In this stage, the CTIP is pushed to the organization, where it is actionable. This is achieved by publishing the CTIP to the corresponding private channel.

## 6. Discussion

The integration of BPC into the CTI domain enables the realization of the proposed approach. In addition, the use of industry standards, including STIX and context schemes for defining the BPC, and MQTT, and the corresponding opensource tool, facilitates broad adoption. The proposed filtering approach was realized via a prototype CTIP sharing ecosystem. During the development of this ecosystem in the lab, the characteristics that an effective CTI filtering approach should meet were identified and documented. Specifically, these characteristics are that filtering should be:

- Performed at the server-side. Performing filtering at the server-side disengages organizations from such a process, allowing them to focus their efforts on processing CTIPs. This saves them from wasting time and resources.
- Based on pushing techniques (rather than pulling). Pushing CTIPs is the second crucial characteristic of filtering. Pushing in conjunction with filtering allows organizations to obtain CTIPs that are actionable to them in a timely manner. Without pushing, organizations have to regularly poll for newly published CTIPs, which is an inefficient computational model.
- Utilizing private communication channels. Private channels allow organizations to privately exchange their sensitive information (e.g., Identity SDO or BPC SDO) as well as CTIPs with the CTIP repository. Private channels also preserve the anonymity of organizations’ actions. For instance, no one can know who published a specific CTIP providing that the Identity SDO of the victim was not included in the CTIP and that the CTIP was published via a private channel. Organizations, however, can include their Identity SDOs in CTIPs to convey the victimization to the community, but this is optional. Such privacy protects organization reputations, promotes trust, and encourages more participation in the ecosystem.

It should also be noted that this paper utilizes the MQTT data transfer protocol, because while the TAXII server was proposed as part of the STIX framework to also satisfy these three characteristics, it has not been realized [34]. For that reason and to explore the potentials of such characteristics, the proposed CTIP sharing ecosystem utilized the MQTT data transfer protocol.

### Related Work

Compared to the proposed approach, there was no other effort found in the literature that offers such a concrete implementation of automated CTIP filtering based on the utilization of BPC. Although there are efforts suggesting the use of business information in CTIP filtering, they lack adequate detail on how it could take shape. For example, SANS did highlight the importance of business information in CTI filtering in [13]. It was proposed that business information consisted of the business context and the technical context. The business context was defined as the business processes, including their dependencies and connections, the data shared among business processes, and the assets supporting business processes (e.g., software, hardware, network devices). The technical context was defined by the past experience and information about adversaries and threats. The business context was used as a filter to identify CTIPs that were relevant to the organization’s processes and the technical context as a filter to identify CTIPs that were relevant to a threat or adversary that an organization might face. Nevertheless, SANS’s approach is hypothetical in the form of suggestions and requires manual and collaborative efforts involving different expert fields, such as CTI and business process experts.

Another example of a similar effort, which is based on SANS, is presented in [23]. That effort presented a model and its corresponding tool that utilized the Euclidean distance to estimate the similarity between different objects based on their properties. This model relied on the business context proposed by SANS to define the organization’s and CTIP’s context. Afterward, it considered the organization’s context and CTIPs as objects. The similarity between these objects results in mapping CTIPs to business processes. This means that these specific business processes risk facing the threats described in the mapped CTIP. However, their business context considered the assets supporting business processes only (e.g., Wifi extender). Other aspects of the business context were not considered nor was the scalability of the approach to additional aspects, particularly related to the similarity estimation.

There are also many efforts in the literature that utilize STIX objects to estimate the relevance between a CTIP and an organization towards achieving filtering. A notable effort is that of [35], which presented a framework and a corresponding tool for estimating the relevance between a CTIP and an organization. The framework utilized the Web Ontology Language to search within STIX objects of a CTIP in order to identify the associated industry sector and location information. If the information matches with that of the recipient, the CTIP is relevant to the recipient. Another notable effort is [21], which also suggested the use of specific characteristics, such as the industry sector in the Identity SDO, that are included in STIX objects to achieve filtering (similar to the domain-tagging approach). In case that a CTIP and an organization share the same characteristics, the CTIP is relevant to the organization. The relevance is further increased by the use of the Sighting SRO. The more times a CTIP has been spotted in the same industry sector, the more relevant it is. It can be observed that all such efforts tried to use business information, but they were limited to those that STIX offers, namely a list of about 35 industry sectors and the location information. Our effort has been shown to address such shortcomings.

There are efforts focusing on domain-tagging of CTIPs in order to present them to organizations operating in the same domain. A notable example of such an effort is [22], which presented the TIMiner framework for automated creation and domain-tagging of CTIPs. TIMiner automatically extracted IoCs (in STIX domain, an IoC is represented by the Indicator SDO) from various locations, such as social media, blogs, and forums, utilizing word embedding and syntactic dependency methods. Afterward, it identified the target domain of these IoCs based on their semantic characteristics and a convolutional neural network deep learning algorithm. Finally, it created CTIPs with these IoCs and tagged them with the identified domain. Using domain-tagging, the TIMiner categorized CTIPs in order to customize their sharing among organizations operating in the same domain. Therefore, TIMiner indeed performed CTIP filtering and sharing by accurately and automatically tagging CTIPs based on their target domain. Nevertheless, TIMiner used arbitrary, high-level domains, such as finance, government, and education, without considering business process-related aspects

## 7. Conclusions

This paper proposed an automated, contextualized cyber threat information filtering approach utilizing the Business Process Context. The BPC is the key enabler for the proposed approach, and it was defined based on standards used in many businesses, including manufacturing. Exploiting standardized and multi-level contextual information of business processes provide improvements over current approaches (e.g., domain tagging) as well as over approaches that use coarse-grained business information in filtering. To enable automation, the Business Process Context was incorporated into the cyber threat information domain as a new user-defined object of the STIX expression language, which is also a standard in the cyber threat information domain. Standards were used in the proposed approach on purpose in order to pave the way for wider adoption.

Utilizing the proposed approach, organizations can receive cyber threat information that is relevant to their environment. This information is expected to be actionable to them in the way that its utilization will result in a decision or an action to control an ongoing, imminent, or future threat. Filtering, therefore, enables organizations to focus their efforts on the actual use of cyber threat information without spending resources and time on excluding irrelevant information. This is of great importance, especially in case of small–medium enterprises that do not possess many resources.

A cyber threat information sharing ecosystem based on open-source tools and protocols was also proposed. It outlined the protocols and techniques that could be used with the proposed approach. Based on the experience implementing the ecosystem, the necessary characteristics that a cyber threat information filtering and sharing system should meet were derived. These characteristics include promoting trust, preserving privacy, and encouraging organizations to participate in cyber threat information sharing. The latter is of critical importance from a forward-looking perspective: the threat landscape is continuously changing and needs increasing collaborations via cyber threat information sharing in order to support early detection and response to emerging threats and ongoing attacks.

Our future research effort will aim at further calibrating the proposed filtering approach and sharing ecosystem in real-world scenarios and conditions, that involves the participation of organizations from different domains and business contexts. Machine Learning algorithms will also be used for comparison against or enhancement of the proposed approach. In addition, future work will aim at applying probabilistic models, such as Bayesian networks, and Machine Learning algorithms, such as convolutional neural network, to discover new potentials in filtering as well as to provide insights on shared cyber threat information via clustering algorithms and Business Process Context. Finally, future works will aim at utilizing information technology-related aspects of business processes, such as the platforms and software operating within them, in the proposed cyber threat information filtering approach.

## Data Availability

Not applicable.
